# CRAC channels and patho-physiology of peripheral organ systems

**DOI:** 10.1042/BST20253062

**Published:** 2025-06-03

**Authors:** Rajesh Bhardwaj, Anant B. Parekh

**Affiliations:** Molecular & Cellular Biology Laboratory, National Institute of Environmental Health Sciences, National Institute of Health, Durham, USA

**Keywords:** calcium signaling, channelopathy, CRAC channels, disease

## Abstract

A rise in cytosolic Ca^2+^ is used as a key signalling messenger in eukaryotic cells. The Ca^2+^ signal drives life and death and controls myriad responses in between. Inherent in the use of such a multifarious signal is the danger of disease, arising from dysregulated Ca^2+^ signalling. One ancient, highly conserved and widespread Ca^2+^ entry pathway is the store-operated Ca^2+^ release-activated Ca^2+^ (CRAC) channel. Mutations in *STIM1* and *ORAI1*, the genes that encode the functional channel, are tightly linked to a CRAC channelopathy in humans, which encompasses severe combined immune deficiency, myopathy and anhidrotic ectodermal dysplasia. Moreover, sustained Ca^2+^ entry through the channels leads to a range of systemic disorders, including acute pancreatitis, asthma and inflammatory bowel disease. In this review, we describe how aberrant CRAC channel activity causes a range of diseases, highlighting commonalities between these diverse pathologies.

## Introduction

Hundreds of hormones, neurotransmitters, paracrine signals and mechanical forces bombard the surface of a mammalian cell each day. These primary stimuli trigger a wide range of responses across an enormous temporal bandwidth, from sub-milliseconds to days. Remarkably, this diverse array of responses is co-ordinated through a surprisingly small number of intracellular second messengers. Of the known second messengers, cytosolic Ca^2+^ is a universal signal used by cells throughout the phylogenetic tree [1,2]. Balanced finely like a tightrope walker, cytosolic Ca^2+^ is both the harbinger of vitality and the spectre of demise. A rise in cytosolic Ca^2+^ is essential for fertilisation of the egg, driving the beginning of life [3]. Throughout life, cytosolic Ca^2+^ regulates neurotransmitter release, muscle contraction, energy production, hormone secretion, cell motility and cell growth and differentiation [3]. However, cytosolic Ca^2+^ can also cause cell death through apoptosis or necrosis [2,3]. An inherent risk of relying on a single signal to control such fundamental physiological processes is that any distortion or compromise of the signal can result in diseases with often debilitating consequences. This review focuses on disorders that arise from aberrant Ca^2+^ signals through one widely distributed Ca^2+^ channel, the store-operated Ca^2+^ release-activated Ca^2+^ (CRAC) channel, drawing on examples from different systems to highlight commonalities.

## A short primer on Ca^2+^ signalling

**Figure 1:**
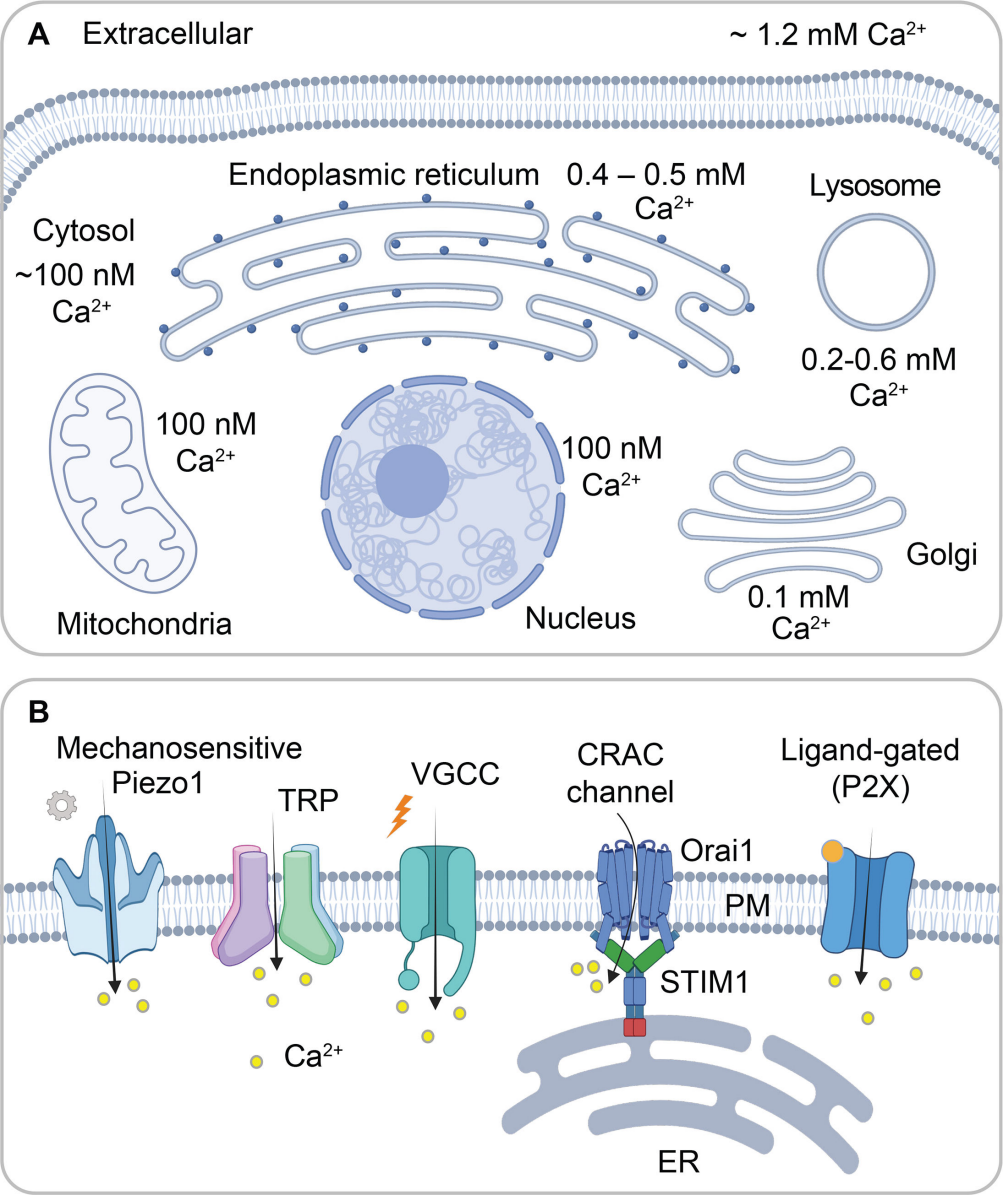
Distribution of calcium across and within cells. (**A**) A cartoon compares the distribution of Ca^2+^ across and within a typical eukaryotic cell. Values shown are for unstimulated conditions. Upon stimulation, Ca^2+^ rises in the cytosol, nucleoplasm and mitochondrial matrix and, depending on the stimulus, falls within the endoplasmic reticulum, lysosome and Golgi apparatus. (**B**) Types of plasma membrane Ca^2+^-permeable ion channels. P2X is an ATP-gated non-selective cation channel representative of the ligand-gated family. The figure was generated using BioRender. TRP, Transient Receptor Potential; VGCC, voltage-gated calcium channel. Values adjacent to organelles represent concentration within the organelle.

The distribution of Ca²^+^ across and within cells is summarised in Figure 1A. The extracellular concentration of free Ca²^+^ is approximately 1.2 mM, while resting cytosolic Ca²^+^ is typically maintained at 50–100 nM. This low cytosolic concentration is maintained by P-type Ca²^+^ ATPase pumps and electrogenic Na^+^-Ca²^+^ exchangers in the plasma membrane. Most cells also have a negative membrane potential, which, combined with the steep Ca²^+^ concentration gradient across the membrane, creates a significant electrochemical gradient that drives Ca²^+^ influx. Because the phospholipid bilayer is impermeable to ions, Ca²^+^ movement occurs through various Ca²^+^-permeable ion channel proteins distributed in the plasma membrane. These channels are gated by a range of factors, including voltage, mechanical forces, temperature and extracellular or intracellular ligands (Figure 1B).

Several organelles function as mobilisable Ca²^+^ stores, including the endoplasmic reticulum, Golgi apparatus and lysosomes [3]. These organelles release Ca²^+^ into the cytosol through Ca²^+^-permeable ion channels in response to specific second messengers. Mitochondria are also heavily involved in cytosolic Ca²^+^ dynamics, taking up Ca²^+^ through the mitochondrial Ca²^+^ uniporter (MCU) and releasing it via Na^+^-Ca²^+^ exchange [4]. However, mitochondrial Ca²^+^ release occurs only after the organelle has accumulated Ca²^+^ through the MCU in response to a prior rise in cytosolic Ca²^+^.

Although Ca²^+^ release from organelles contributes to cytosolic Ca²^+^ signalling, the limited storage capacity of these organelles means that influx from the large extracellular Ca²^+^ reservoir is essential to sustain Ca²^+^ signalling. Furthermore, the substantial electrochemical gradient for Ca²^+^ entry makes an increase in membrane permeability following the opening of a Ca²^+^ ion channel highly effective in rapidly raising cytosolic Ca²^+^. One major type of Ca²^+^ channel is the store-operated CRAC channel.

## CRAC channels: an ancient and conserved route for Ca^2+^ influx

CRAC channels in the plasma membrane comprise an evolutionarily conserved and widespread route for Ca^2+^ entry [5,6]. These channels are robustly expressed in electrically non-excitable cells, such as immune cells, epithelia, hepatocytes, endothelia and glia but are also found in neurons and muscle, although their function in excitable cells remains less well established.

The defining feature of CRAC channels is their dependence on the Ca^2+^ content of the endoplasmic reticulum (ER) [7]. When the Ca^2+^ store is full, the channels are closed, but when Ca^2+^ within the store falls, the channels open. Regardless of how stores are depleted of Ca^2+^, the channels activate. Physiologically, this is generally accomplished following activation of G protein-coupled receptors that link to Gq heterotrimeric GTP-binding proteins and phospholipase Cß isoforms or growth factor receptors that activate phospholipase Cγ enzymes. In both cases, phospholipase C hydrolyses the membrane phospholipid phosphatidyl inositol 4,5-bisphosphate to generate the second messengers inositol 1,4,5-trisphosphate (InsP_3_) and diacylglycerol. Diacylglycerol stimulates protein kinase C, whereas InsP_3_ diffuses into the cytosol and rapidly releases Ca^2+^ by binding to and opening InsP_3_-gated Ca^2+^ channels in the ER membrane [3].

**Figure 2:**
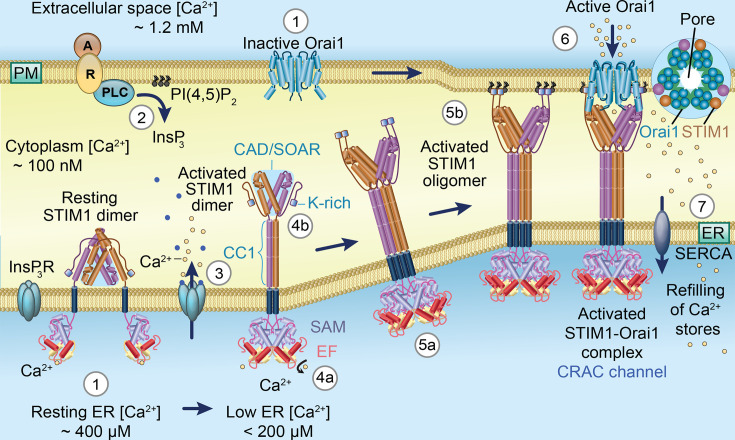
Molecular basis of the Ca^2+^ release-activated Ca^2+^ (CRAC) channel. In resting cells with endoplasmic reticulum (ER) replete with Ca^2+^ (~400 µM), STIM1 dimers and Orai1 channels are in inactive states in the ER and plasma membrane, respectively (1). Stimulation of cell-surface receptors increases InsP_3_ (2), and this leads to Ca^2+^ store depletion (3), where ER Ca^2+^ content falls significantly. The drop in lumen Ca^2+^ results in Ca^2+^ disassociation from the EF hand of STIM1 (4a). Unbinding of Ca^2+^ activates STIM1 dimers by promoting aggregation and interaction of EF-SAM domains within the ER lumen (4a), extending the coiled coil 1 (CC1) domain and exposing the locked STIM1 cytosolic channel-activating domain/STIM Orai-activating region (CAD/SOAR) region and the PI(4,5)P_2_ binding lysine (K)-rich domain (4b). STIM1 forms oligomers, which bind the phospholipid PI (4.5)P_2_ at the inner leaflet of the plasma membrane via the lysine (K)-rich domain (5b). This stabilises pre-existing ER-plasma membrane junctions and forms new ones. Exposed CAD/SOAR binds to the C-terminus of diffusing Orai1 channels, trapping them at the junctions and gating them open (6). SERCA pump activity, together with a fall in InsP_3_-dependent Ca^2+^ release, enables store refilling of Ca^2+^ (7). Ca^2+^ then reassociates with the EF hand of STIM1, leading to disaggregation of the STIM1-Orai1 complex and termination of Ca^2+^ influx (1). Adapted and modified from [12].

The CRAC channel machinery is a multi-component system comprising two key players: Orai and STIM proteins. Orai proteins, located in the plasma membrane, form the pore-lining subunits of CRAC channels [8–11] (Figure 2). STIM proteins, embedded in the ER membrane, act as Ca^2+^ sensors that regulate CRAC channel gating and activity [13,14] (Figure 2). These proteins have a canonical Ca²^+^-binding EF hand on the N-terminus facing the ER lumen. In humans, three Orai paralogs (Orai1, Orai2 and Orai3) and two STIM paralogs (STIM1 and STIM2) are present, which, along with several splice variants, alternative translation initiation forms of Orai1, and differences between STIM1 and STIM2 in Ca^2+^ affinities, enable more precise fine-tuning of store-operated Ca^2+^ entry [5,6]. Following a physiological stimulus, Ca^2+^ levels in the ER lumen drop as a result of InsP_3_-dependent Ca²^+^ release. The fall in ER Ca^2+^ content is detected by STIM1 and STIM2 through Ca^2+^ unbinding from the EF hand domains. This process induces a conformational change that unmasks cytosolic domains, resulting in exposure of channel-activating regions (Figure 2). STIM proteins then translocate across the ER to specialised regions that are located just below the plasma membrane. Here, the exposed channel-activating domain, also called STIM Orai-activating region of the activated STIM proteins, interacts with the Orai subunits to assemble and gate open the pore, allowing the influx of extracellular Ca^2+^ through CRAC channels [15,16] (Figure 2).

X-ray crystallographic studies on *Drosophila Orai* revealed that the channel pore is composed of a hexameric ring of Orai proteins, each with four transmembrane (TM) helices, where six TM1 helices, one from each subunit, form the pore [17]. Numerous studies have investigated the gating rearrangements in Orai1 that enable the channel pore to transition between closed and open states following STIM1-mediated binding, and various residues and segments on both Orai1 and STIM1 have been identified as critical for channel activation and inactivation [18,19]. A wide range of mutations, including disease-causing gain-of-function (GoF) mutations, have been reported in Orai1, enabling constitutive Ca^2+^ entry independent of store depletion and STIM1 activation. While the majority of GoF mutations studied compromise the selectivity of the Orai channel for Ca^2+^ ions, mutations at only two loci—one in TM2 (H134) and another in the TM4 cytosolic extension (LVSHK)—have been suggested to mimic the STIM1-gated state of the channel. The TM4 region of human Orai1 features a kink at the conserved proline residue (P245) and a cytosolic ‘nexus’ sequence (LVSHK) crucial for channel activation. Early studies on *Drosophila melanogaster* Orai described the TM4 extension as being locked in a ‘latched’ state, preventing pore opening, but the physiological relevance of this state remains contested. Mutations in various Orai regions, including pore-lining residues such as F99 and V102, cause constitutive channel opening by altering gating mechanisms, while mutations in TM2 (e.g. H134) and TM4 can trigger conformational changes leading to channel activation [18,20]. Future structural studies exploring both the closed and open states of the Orai channel in complex with STIM1 are expected to address these unresolved questions on the gating mechanisms of CRAC channels in health and disease.

Orai1 participates in a reversible signalosome with AKAP79. AKAP79 is a scaffolding protein embedded in the inner leaflet of the plasma membrane and acts as a signalling hub through binding protein kinases A and C, calmodulin, as well as the Ca^2+^-activated protein phosphatase calcineurin [21]. Activated calcineurin dephosphorylates four members of the transcription factor nuclear factor of activated T cells (NFAT1-4), exposing a nuclear localisation sequence, which enables the transcription factor to translocate to the nucleus, where it regulates the expression of various cytokines and chemokines that help shape an inflammatory response. After store depletion, AKAP79 associates with the N-terminus of Orai1, thereby placing the Ca^2+^-activated calcineurin within the realm of the local Ca^2+^ signal adjacent to the open channels [22]. This robustly activates the phosphatase and leads to dephosphorylation and subsequent nuclear translocation of NFAT. Intimate functional association between Orai1 and AKAP79 enables CRAC channels to have privileged access to the nucleus. CRAC channels thus not only engage local Ca^2+^ signalling to drive downstream Ca^2+^-dependent processes but also sustain Ca^2+^ signalling by enabling Ca^2+^ store refilling via the action of SERCA pumps.

## CRAC channelopathy

Important insight into the function of STIM and Orai1 proteins has been gleaned from studies on patients suffering from immunodeficiencies with loss-of-function (LoF) mutations in these genes. Although very rare, all the mutations produce a similar set of phenotypes, leading to what Stefan Feske has called the CRAC channelopathy [23]. The channelopathy encompasses immunodeficiency, exemplified by recurrent severe infections to a broad range of pathogens, including viruses, bacteria, mycobacteria and fungi. These infections can lead to death shortly after birth unless haematopoietic stem cell transplantation is provided. The immunodeficiency was traced to an inability of CD4+ and CD8+ T cells to proliferate and produce cytokines [24]. In the absence of store-operated Ca^2+^ entry through Orai1, NFAT activation is prevented, and this contributes to impaired cytokine production. The patients also present with myopathy, exhibiting global muscle hypotonia with reduced muscle strength, and anhidrotic ectodermal dysplasia manifested as reduced sweat production and loss of dental enamel [25]. Most of the patients with LoF mutations in STIM1 also show thrombocytopenia and autoimmune haemolytic anaemia. Interestingly, thrombocytopenia was less common in patients with LoF mutations in Orai1 [25].

## Diseases

### GI tract

#### Acute pancreatitis

**Figure 3:**
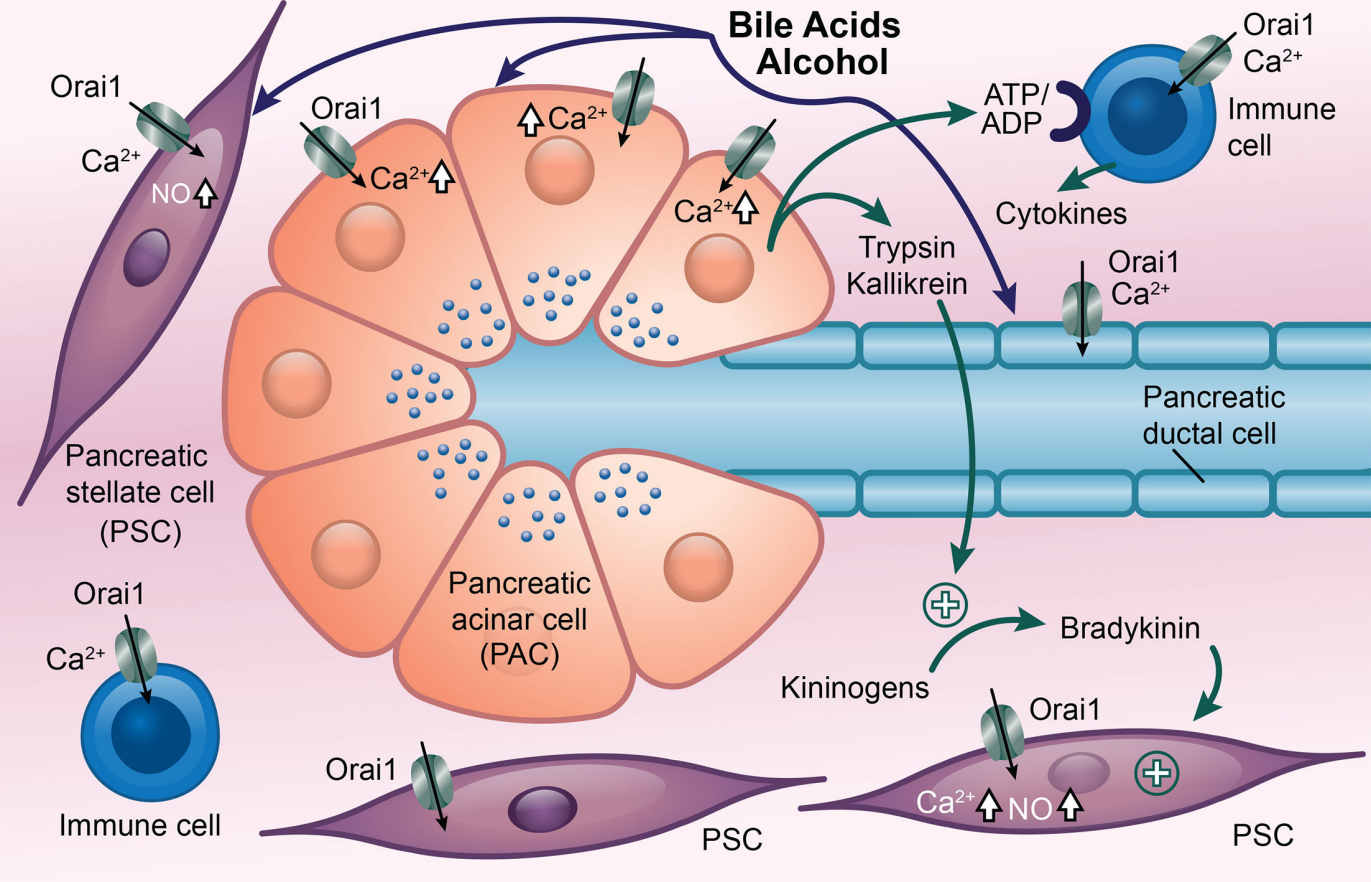
Orai1 channels play a central role in the development of acute pancreatitis. Sustained Ca^2+^ entry through Orai1 channels in PACs following stimulation with bile acids or alcohol leads to premature activation and secretion of trypsin, which autodigests the pancreas. Damaged PACs also release other signals, including kallikrein and adenine nucleotides. Kallikrein cleaves kininogens to generate bradykinin, which acts on bradykinin type 2 receptors in PSCs to open Orai1 channels. This generates nitric oxide, which diffuses to PACs and promotes necrosis. ATP/ADP released from PACs activates Orai1 channels in immune cells, which leads to a local inflammatory response through the release of cytokines. Blue circles at apical pole denote trypsin-containing granules, which are normally released into the pancreatic duct.

Acute pancreatitis is caused by inflammation of the pancreas, typically triggered by excessive alcohol consumption, bile duct obstruction (e.g. due to gallstones), certain medications (including thiazides, tetracycline, sulfonamides and the immunosuppressant azathioprine), pancreatic cancer, elevated blood triglycerides or pancreatic trauma resulting from injury or surgery [26]. The condition arises from inappropriate activation of trypsinogen and other proteolytic proenzymes that are stored within zymogen granules in the apical pole of pancreatic acinar cells (Figure 3). These proteases then autodigest the pancreas, resulting in necrosis [27]. Agents that trigger acute pancreatitis, such as alcohol and fatty acid ethyl esters, induce a sustained elevation of cytosolic Ca^2+^ due to Orai1-based CRAC channel opening, which leads to the activation of trypsin within endocytic vacuoles and mitochondrial Ca^2+^ overload, disrupting ATP production (Figure 3) [28,29]. Pharmacological block of the CRAC channel with GSK-7975A suppressed Ca^2+^ entry and significantly decreased both intracellular protease activation and necrosis induced by palmitoleic acid ethyl ester. In mouse models of acute pancreatitis, the Orai1 channel inhibitors GSK-7975A and CM_128/CM4620 independently suppressed local and systemic pathophysiology [30] and prevented recurrent acute pancreatitis and early stages of chronic pancreatitis [31]. A phase 2 study on 21 patients with acute pancreatitis, systemic inflammatory response syndrome and hypoxemia showed a favourable safety profile and outcomes in patients treated with a CRAC channel blocker [32].

Although effective, CRAC channel blockers do not fully prevent acute pancreatitis even at high doses. A recent study reported that the combination of CM4620 and galactose, an energy supplement that restores ATP production, was more effective than CM4620 alone [33]. Moreover, the combination allowed for a significantly lower dose of CM4620 to be used, reducing potential off-targets arising that could be induced by a higher dose of drug [33].

CM4620 reduced the severity of acute pancreatitis in a rat model but did so not only by targeting the pancreatic acinar and stellate cells in the pancreas but also by reducing the neutrophil oxidative burst and the release of inflammatory mediators from invading immune cells [34], although Orai1 in macrophages might not be involved [35]. The pancreatic duct transports enzymes secreted from acinar cells to the small intestine, and ductal cells secrete bicarbonate-rich fluid to neutralise acidity in the gut from H^+^ secretion by parietal cells in the stomach. Ca^2+^ overload in ductal cells would compromise function and reduce the removal of digestive enzymes from the duct, exacerbating pancreatic autodigestion. CM5620, another CRAC channel blocker, reduced Ca^2+^ influx into pancreatic ductal cells in response to bile acids or ethanol challenge, protecting secretory ductal function. CRAC channel block also partially maintained pancreatic secretion in a mouse model of cerulein-induced pancreatitis [36]. Therefore, a CRAC channel blocker targets multiple cell types associated with acute pancreatitis at the same time (Figure 3).

Another potential approach could target CRAC channels indirectly. Store-operated calcium entry-associated regulatory factor (SARAF) is an ER-resident protein that negatively regulates CRAC channel activity. Expression of SARAF was found to fall in mice exposed to triggers of pancreatitis and in patients with pancreatitis [37]. Mice in which the SARAF gene had been knocked out developed more pronounced pancreatitis, whereas overexpression of SARAF suppressed inflammation and dampened pancreatitis [37].

#### Inflammatory bowel disease (IBD)

Inflammatory bowel disease (IBD) is characterised by chronic inflammation of the digestive tract, with the two main manifestations being ulcerative colitis and Crohn’s disease. Although idiopathic in nature, IBD is thought to arise from a combination of genetic and environmental factors, with the latter involving the gut microbiome, surgery, smoking or exposure to a range of early life factors, including GI infections and antibiotics. Prolonged IBD is associated with the development of colorectal cancer. A hallmark of IBD is a dysregulated intestinal immune response, particularly within the lamina propria. T cells isolated from human inflamed mucosa showed substantially larger store-operated Ca^2+^ entry and proliferation, when compared with those extracted from non-inflamed tissue [38]. This can be explained by the ~fivefold increase in STIM1 protein expression in human inflamed intestinal mucosa with significant staining in CD45+ cells within the lamina propria [39]. The CRAC channel inhibitor Synta66 reduced the levels of inflammatory cytokines IFN-γ, IL-2 and IL-17 in biopsy specimens from patients with IBD, pointing to an important role for CRAC channels in immune cell activation [40]. The immunosuppressant FK506, which would inhibit all CRAC channel-dependent gene expression through NFAT activation, also reduced IFN-γ, IL-2 and IL-17 levels to a similar extent to Synta66. However, FK506 also suppressed IL-8 expression, whereas Synta66 was ineffective [41]. The reason for this difference remains unclear. Genetic evidence for a role for CRAC channels was provided in a study that generated Orai1 knock-in mice expressing inactive R93W (human R91W) channels. These channels are expressed normally but cannot be gated by STIM proteins and, therefore, do not conduct Ca^2+^. T cells expressing the R93W mutant channels failed to induce colitis in an adoptive transfer model [41]. A detailed study utilising mass cytometry found an increased number of naïve T cells, CD4+ T cells, IFN-γ-producing CD8+ T cells, T-regs and innate lymphoid cells in the lamina propria of inflamed intestinal tissue from IBD patients [42]. The CRAC channel blocker BTP2 inhibited store-operated Ca^2+^ entry and reduced the production of multiple pro-inflammatory cytokines. Importantly, BTP2 had no effect on intestinal epithelia cell differentiation or on barrier function, demonstrating a beneficial effect on immune cells without compromising epithelial cell activity or network integrity. Another CRAC channel blocker, CM4620, was found to be more potent than BTP2 and was effective in reducing intestinal inflammation and subsequent damage in a mouse model of IBD [42].

### Lung

#### Asthma

Asthma is a chronic inflammation of the lung that results in prolonged bouts of coughing, tightness of the chest, wheezing and shortness of breath. Asthma is a heterogeneous disease that can be driven by both genetic and environmental factors [43]. Sources of environmental aero-allergen include those released by the house dust mite, cockroach, fungi, animal dander and pollen. The house dust mite is the major source of aero-allergens in humans, and sensitisation to mite-derived allergens is a common cause of asthma [44,45]. The main types of house dust mite are *Dermatophagoides pteronyssinus* and *Dermatophagoides farina*, with allergens derived from them referred to as Der p and Der f proteins. Many mite-derived allergens are enzymes, particularly cysteine and serine proteases [44].

**Figure 4:**
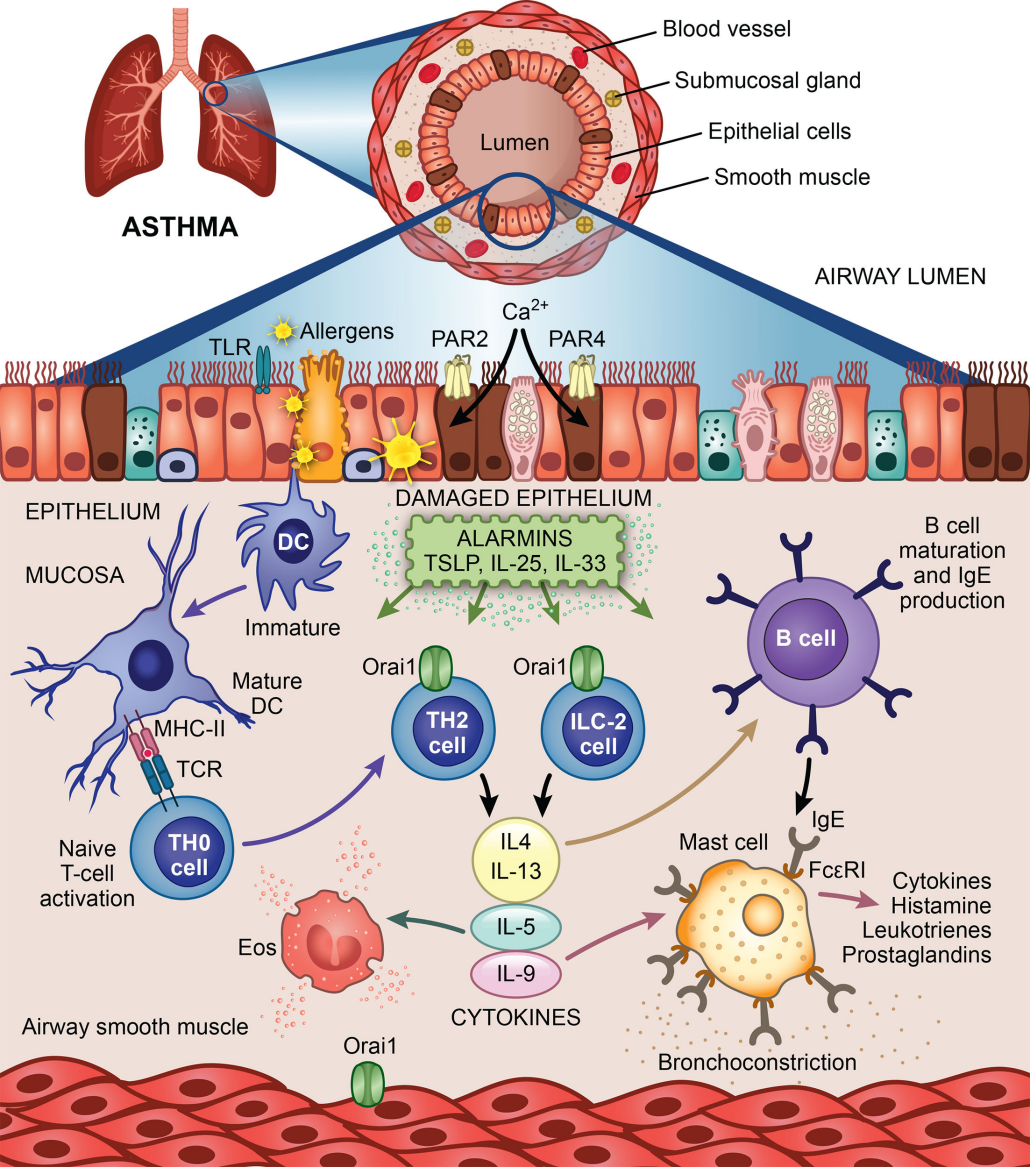
CRAC channels and asthma. Interplay between airway epithelia, smooth muscle and immune cells leads to the development of chronic inflammation in lungs following exposure to allergens. Immature dendritic cells, after being exposed to allergens and alarmins, travel to draining lymph nodes where they activate naïve CD4+ T cells, which then migrate to the lung. TLR denotes toll-like receptor; TSLP is thymic stromal lymphoprotein; DC is dendritic cell; Eos is eosinophil; and ILC-2 is innate lymphoid type 2 cell. See text for further details. Not all immune cells are displayed for simplicity.

Inhaled allergens stimulate airway epithelia to release alarmins from their basolateral side, which then activate various lung-resident immune cells, including innate lymphoid type 2 (ILC2) cells, dendritic cells and mast cells (Figure 4) [44–47]. Released cytokines, chemokines, enzymes and paracrine signals recruit other immune cells, including Th2 lymphocytes and IgE-producing B cells, contract airway smooth muscle and drive remodelling of the airways (Figure 4). Meanwhile, allergens weaken the integrity of the epithelial barrier, damaging tight junctions and enabling their passage through the paracellular pathway. STIM1 is up-regulated in airway smooth muscle of asthmatic mice and is required for the metabolic and transcriptional reprogramming that is necessary for remodelling [48]. Whether store-operated Ca^2+^ entry contributes to epithelial barrier function is currently not known.

Exposure of airway epithelial cells, mast cells or Jurkat T lymphocytes to mite extract resulted in Ca^2+^ entry through CRAC channels, which led to increased expression of pro-inflammatory genes and cell migration [49,50]. These responses were accomplished through activation of protease-activated receptors (PARs) type 4 and 2 (PAR4 and PAR2). PARS are Gq-coupled receptors that stimulate phospholipase C to generate InsP_3_. InsP_3_ releases Ca^2+^ from the ER into the cytosol, lowering store Ca^2+^ content and leading to subsequent gating of Orai1 by STIM1 through the canonical pathway. CRAC channel blockers or knockdown of STIM1 or Orai1 inhibited the sustained Ca^2+^ signal evoked by house dust mite, as well as gene expression and cell migration [49,51]. In airway epithelia, CRAC channel activation leads to NFAT signalling and subsequent expression of pro-inflammatory cytokines IL-6 and IL-8 [49], as well as c-fos activation [51]. CRAC channels are also required for epithelial cell migration [51].

House dust mite extract contains a plethora of allergens along with adjuvants, lipids and other signalling molecules. Combining cytosolic Ca^2+^ measurements with extract fractionation and mass spectrometry, Lin et al. identified Der p3 as a critical component in the mite extract that activated CRAC channels [51]. Der p3 is a serine protease and stimulates PAR4. Like other serine proteases, inactive pro-Der p3 is cleaved by the cysteine protease Der p1 to release active Der p3. The combination of recombinant Der p1 and pro-Der p3 mimicked the ability of dust mite extract to open CRAC channels. Targeting both CRAC channels and PAR4 pharmacologically at the same time, with sub-maximal concentrations of each inhibitor, suppressed house dust mite-induced activation of mast cells [51]. These *in vitro* studies suggest that targeting Der p3, PARs and CRAC channels should open up new targets for intervention, alone or in combination.

Several studies have demonstrated the efficacy of CRAC channel blockers in dampening the severity of allergic asthma in various models. BTP2 inhibited cytokine release and eosinophil infiltration in rat and guinea pig models of asthma [52]. Synta66 substantially decreased cytokine release from lymphocytes that had been isolated from bronchioalveolar lavage of healthy and asthmatic donors [53]. The pyrazole RP3128 reduced eosinophil and mast cell infiltration following antigen challenge of sensitised guinea pigs [54]. In a mouse model of house dust mite-induced asthma, oral application of the CRAC channel blocker CM4620 significantly reduced the severity of the asthmatic response [55]. Peribronchial inflammation, mucus production, levels of pro-inflammatory cytokines (IL-4, IL-5, IL-13 and IL-16) were all reduced following CRAC channel inhibition, as were the number of B cells, eosinophils and CD4+ T cells found in bronchoalveolar lavage fluid [55].

Group 2 ILC2s are activated by various alarmins from airway epithelia to release type-2 cytokines that help drive lung inflammation (Figure 4). Orai channels in ILC2 cells are important for the development of airway hypersensitivity and inflammation in the lung [56]. Challenge with the fungus *Alternaria alternata* increased airway resistance, as well as the number of eosinophils in bronchoalveolar lavage fluid, and these responses were less pronounced in the presence of a CRAC channel blocker [56]. Importantly, adoptive transfer of Orai1^-/-^ or Orai1/2^-/-^ ILC2 cells reduced airway hypersensitivity and eosinophilia compared with control ILC2 cells [56].

Collectively, these experiments provide strong evidence for the role of CRAC channels in asthma in an *in vivo* model and reinforce the view that targeting the channels should be an effective treatment for managing the condition.

### Teeth

#### Enamel

One of the hallmarks of patients with a CRAC channelopathy is ectodermal dysplasia, which results in severely dysplastic dental enamel [23,41,57]. Enamel is produced by ameloblasts, which secrete proteins, such as enamelin and amelogenin, that mineralise and form hard enamel. Specific deletion of *STIM1/2* genes in murine ameloblasts resulted in diminished store-operated Ca^2+^ entry and a hypomineralised enamel with a lower Ca^2+^ content, which was thinner and mechanically weaker than normal enamel [58]. The loss of STIM proteins led to an increase in reactive oxygen species, reduced mitochondrial function, altered mitochondrial shape and a redistribution of the organelle within the cytosol. Detailed investigation into a patient homozygous for a frameshift mutation and subsequent premature stop codon in TM3 of Orai1 (V181SfsX8) confirmed loss of store-operated Ca^2+^ entry [59]. The patient presented with pitted enamel hypoplasia of the primary central incisors and hypomineralisation of the primary maxillary incisors [59]. Ca^2+^ entry into ameloblasts through Orai1, therefore, plays a central role in enamel production. Potential effects of Orai1 inhibitors on enamel production will need to be carefully monitored, particularly in children when such compounds are used in the clinic.

### Sweat glands

#### Anhidrosis

**Figure 5:**
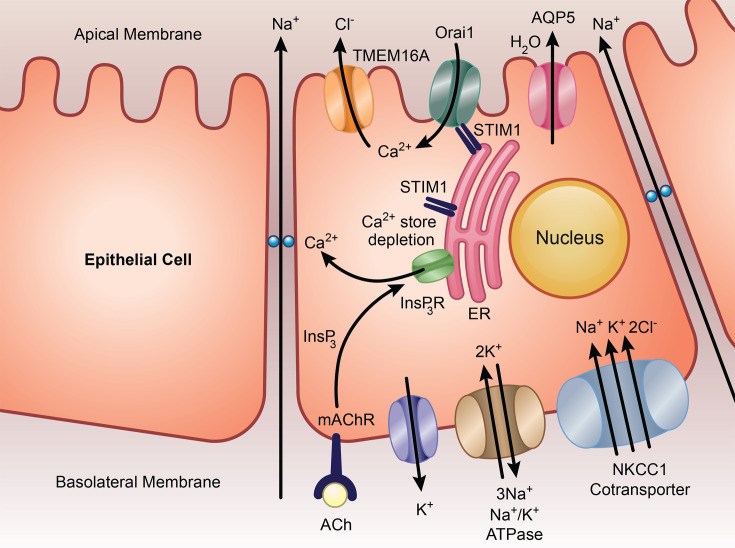
Salt secretion in eccrine sweat glands requires Orai1. Stimulation of muscarinic receptors on the basolateral membrane increases InsP_3_, leading to Ca^2+^ release from the ER. Store depletion opens Orai1 channels in the apical membrane. Ca^2+^ entry through the channels stimulates Ca^2+^-activated Cl^-^ channels (TMEM16A), which results in Cl^-^ secretion across the apical membrane. Cl^-^ levels in the cytosol are maintained by electroneutral Na^+^- and K^+^-coupled Cl^-^ co-transport (NKCC1) in the basolateral membrane. Na^+^ is expelled by the Na^+^-K^+^ ATPase pump, and the increase in cytosolic K^+^ then exits through basolateral K^+^ channels. Na^+^ is secreted transcellularly through the paracellular pathway, driven by the small negative potential in the lumen of the gland (apical side). Water follows through aquaporin channels.

Eccrine sweat glands are found dispersed throughout human skin, where they play an important role in the regulation of sweating and thermoregulation. Patients with LoF mutations in STIM1 or Orai1 exhibit marked anhidrosis [23]. In normal sweat glands, Ca^2+^ entry through Orai1 channels activates TMEM16A, a Ca^2+^-activated Cl^-^ channel in the plasma membrane (Figure 5). Cl^-^ efflux from the cell provides the driving force for water flow through aquaporins.

In mice, eccrine sweat glands are found only on the foot pads. Cholinergic stimulation of the hind paws resulted in an increase in the number of open sweat pores in wildtype mice, but very few opened in animals in which Orai1 had been deleted in tissues of ectodermal origin, including sweat glands [60]. Interestingly, sweat glands had normal morphology in the Orai1 knockout mice, but each lumen was narrower. The finding that patients with LoF mutations in Orai1 present with anhidrosis suggests that an intimate association between Orai1 and TMEM16A exists, because other sources of Ca^2+^ cannot substitute for Orai1.

### Skin

#### Psoriasis

Psoriasis vulgaris is a chronic inflammatory skin disorder that affects ~2–4% of the global population and manifests as marked plaque-like skin lesions that significantly reduce quality of life. The lesions arise from epidermal hyperplasia and immune cell infiltration, but the mechanisms involved are only partly understood. Psoriasis is driven, in part, by a Th-17 specific immune response [61]. Dendritic cells and keratinocytes overproduce IL-23, which then stimulates various cell types within the dermis, including Th-17 cells, mast cells and macrophages. These cells then release pro-inflammatory cytokines, including IL-17A and TNF-α, which trigger keratinocyte hyperproliferation, as well as secretion of chemokines that recruit neutrophils. Keratinocytes and skin immune cells express STIM and Orai proteins, and Orai1 is required for keratinocyte proliferation and polarised motility [62]. The CRAC channel blocker BTP2 inhibited contact hypersensitivity to the dermatitis-inducing allergen TNCB, as well as delayed type hypersensitivity to sheep red blood cells [63], which is often used as a model of dermatological autoimmune disease. Two CRAC channel blockers were reported to reduce serum cytokine levels and psoriatic plaques in a mouse model of psoriasis [64].

In another study, pathogenic Th17 cells were generated in mice following T cell-specific expression of a hyperactive form of the transcription factor STAT3C [65]. This led to psoriasis-like skin inflammation, hyperkeratosis and psoriatic arthritis. Importantly, these phenotypes were prevented by STIM1 deletion in T cells, resulting in a loss of store-operated Ca^2+^ entry [65]. Therefore, CRAC channels are required for Th17-dependent psoriatic lesions.

### Autoimmune disease

#### Primary Sjögren’s disease

Primary Sjögren’s disease is an autoimmune disease that affects 0.5–1% of the population and disproportionately affects females [66]. Hallmarks include exocrinopathy, leading to dryness of the mouth and eyes due to dysfunction of salivary and lacrimal glands, general fatigue and joint pain. In primary Sjögren’s syndrome, the salivary and lacrimal glands exhibit prominent inflammation, lymphocyte infiltration and ultimately are damaged to the extent that function is heavily impaired [66].

A mouse with conditional knockout of *STIM1* and *STIM2* genes in T cells showed spontaneous and progressive submandibular gland inflammation, typically within 3 months, marked salivary gland damage, loss of stimulated fluid secretion, lymphocyte invasion of the glands and elevated primary Sjögren’s disease-specific autoantibodies [67]. Peripheral blood mononuclear cells from patients with primary Sjögren’s disease had reduced STIM protein expression and substantially reduced store-operated Ca^2+^ entry compared with healthy volunteers [67]. It was suggested that dysregulation of STIM protein expression with subsequent aberrations in store-operated Ca^2+^ entry in T lymphocytes underlies the pathogenesis of primary Sjögren’s syndrome [67]. A recent study used a K14Cre driver to specifically delete STIM1 and STIM2 in the ectoderm and its derivatives, which includes salivary glands [68]. Store-operated Ca^2+^ entry was decreased in parotid acini from STIM1/2-deficient mice. In the acini, store-operated Ca^2+^ entry activates ANO1, a Ca^2+^-activated Cl^-^ channel, resulting in Cl^-^ secretion into the lumen of the gland. This generates a negative electrical potential, which draws Na^+^ into the lumen through the paracellular pathway and water through aquaporin 5 channels. The reduction in store-operated Ca^2+^ entry in the STIM-deficient animals, therefore, resulted in less Cl^-^ and Na^+^ secretion, and consequently less saliva production compared with controls. It is important to note that in this study [68], STIM had been deleted in secretory epithelial cells and not T cells. Therefore, the work is more relevant to non-autoimmune Sicca’s syndrome but not Sjögren’s disease. A recent rigorous study has demonstrated that regulatory T cells (Tregs) are particularly important in this context [69]. Deletion of STIM1/2 specifically from Foxp3+ Tregs in mice fully recapitulated all the features of primary Sjögren’s disease [69]. The animals showed salivary and lacrimal gland inflammation, marked lymphocyte infiltration and increased autoantibodies in serum, and met all classification criteria for human disease. The disease was caused by interferon gamma-producing CD4+ T cells. Inhibition of interferon signalling through application of a JAK1/2 inhibitor reduced CD4+ T cell-induced Sjögren’s disease [69].

### Skeletal muscle

#### Tubular aggregate myopathy and Stormorken’s syndrome

**Figure 6:**
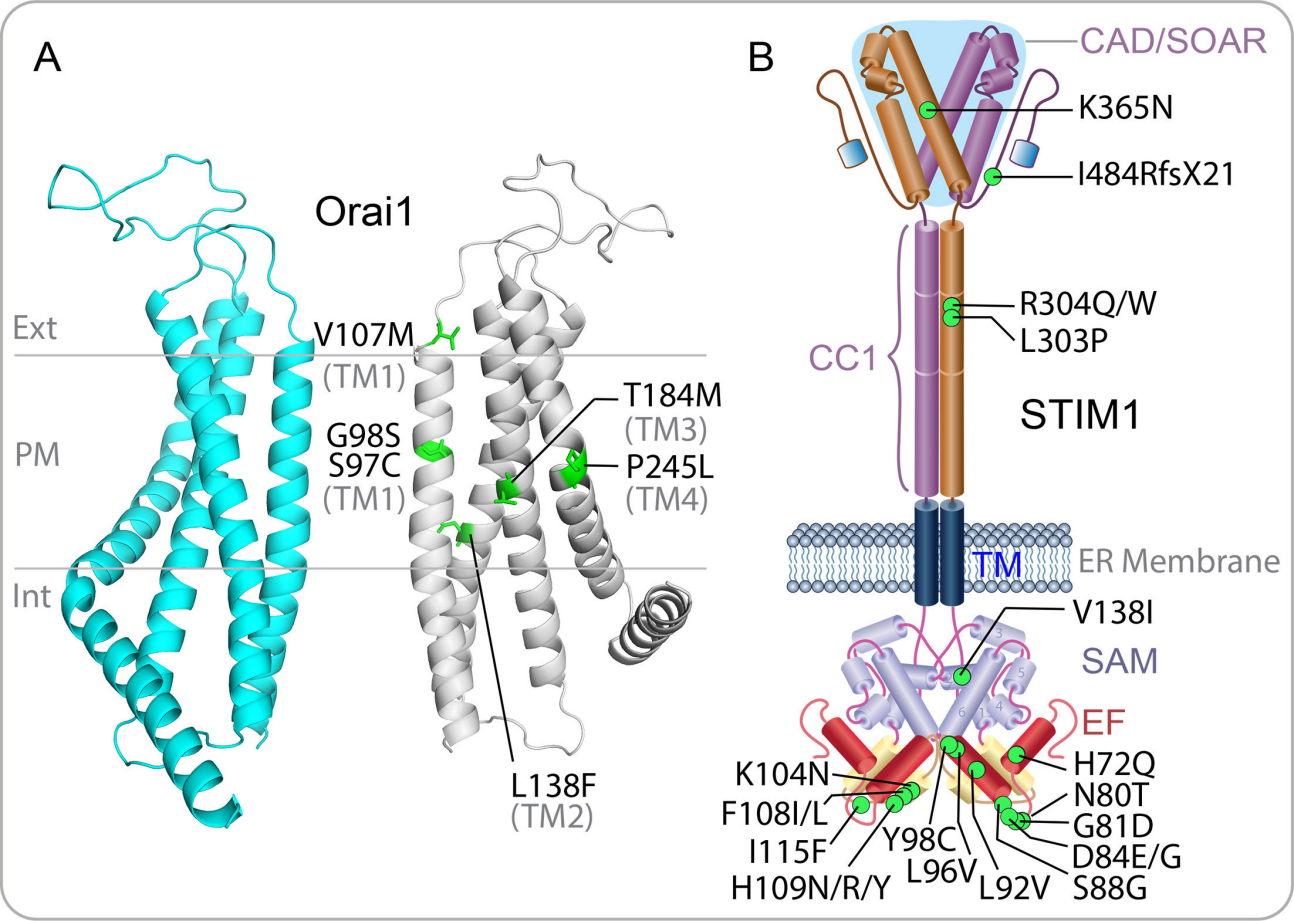
Human Orai1 and STIM1 GoF mutations in TAM are shown. Human Orai1 model was modified from [20]. Only two Orai1 subunits are shown for simplicity. GoF, gain-of-function; TAM, tubular aggregate myopathy. PM plasma membrane, Ext. extracellular, Int intracellular.

CRAC channels play a crucial, albeit indirect, role in supporting skeletal muscle contraction through preventing fatigue, regulating metabolism and supporting muscle development, growth and regeneration. Several dominant GoF mutations in Orai1 and STIM1 have been identified in patients with tubular aggregate myopathy (TAM) and Stormorken syndrome (STRMK) [70]. TAM is primarily characterised by progressive muscle weakness, exercise intolerance and tubular aggregates (TAs). The latter are formed by densely packed tubules derived from the sarcoplasmic reticulum (SR) in skeletal muscle. In contrast, STRMK presents a broader multisystemic disorder, including thrombocytopenia, miosis, hypersplenism, ichthyosis, short stature and dyslexia, alongside muscle weakness and TAs. Six mutations in the transmembrane domains of Orai1 (S97C, G98S, V107M, L138F, T184M and P245L) and 22 in different STIM1 domains [71] (H72Q, N80T, G81D, D84E/G, S88G, L92V, L96V, Y98C, K104N, F108I/L, H109N/R/Y, I115F, V138I, L303P, R304Q/W, K365N and I484RfsX21) have been found in TAM/STRMK patients (Figure 6). These STIM1/Orai1 GoF mutations result in constitutive Ca²^+^ entry and enhanced store-operated Ca^2+^ influx, even in the absence of SR Ca²^+^ store depletion. The most prevalent STIM1 mutation, R304W, has been observed in STRMK patients from 12 unrelated families, and corresponding knock-in mouse models suggest a central role for dysregulated Ca^2+^ entry in disease aetiology [72,73]. While most known STIM1 TAM/STRMK-linked mutations affect STIM1 activation by releasing the conformational lock and resulting in Orai1-mediated Ca^2+^ entry even under resting conditions, one exception involves a STIM1 frameshift mutation (I484RfsX21) that lifts the inactivation brake of the CRAC channel [74]. The TAM-associated STIM1^I484R^ mutation increases ORAI1 channel function due to a reduced STIM1 inactivation break and an absence of microtubule trapping. None of the STIM1 TAM/STRMK GoF mouse models (*Stim1*^R304W/+^, *Stim1*^I115F/+^ and *Stim1*^D84G/+^) exhibit TAs, despite displaying other clinical hallmarks such as muscle weakness [71]. However, the very recently developed Orai1 TAM mice models (*Orai1*^G100S/+^ and *Orai1*^V109M/+^), equivalent to human G98S and V107M, showed the presence of TAs along with muscle weakness [75,76]. Constitutive Ca²^+^ entry was observed in muscle cells during early development of *Orai1*^G100S/+^ mice but was abolished in adulthood; store-operated Ca^2+^ entry (SOCE) was significantly reduced, likely due to decreased Orai1 expression, and mitochondrial dysfunction was also observed, possibly linking Orai1 activity to mitochondrial damage.

While the precise mechanism of TAs formation remains unclear, these structures may play a protective role in TAM/STRMK muscle by trapping misfolded proteins and excess Ca^2+^, thereby reducing cellular stress and preventing myofibre damage [70]. However, caution is needed when analysing muscle phenotypes in transgenic mice, as TAs, identical with those in human diseases, can be naturally found in muscle fibres of male inbred mice and are influenced by age and sex [77]. Mutations in CASQ1 and RYR1 have also been linked to TAM [71].

Orai1 has garnered significant attention as a major therapeutic target for TAM/STRMK. Decreasing the expression of Orai1 in Stim1^R304W/+^ mice by crossing it with Orai1^+/-^ mice improved the multi-systemic TAM/STRMK phenotype [78]. Another recent study showed that crossing the STIM1^R304W/+^ mouse model with the partly obstructed ORAI1^R93W/+^ variant improved muscle function and alleviated multisystemic abnormalities [79]. *In cellulo*, the constitutive Ca^2+^ influx through most of the human Orai1 TAM/STRMK mutants (except the G98S Orai1) could be blocked by sub-micromolar concentrations of the CRAC channel inhibitor GSK-7975A [80]. Newly discovered SOCE modulators, CIC-37 and CIC-39, were able to inhibit Ca^2+^ influx through various STIM1/Orai1 GoF TAM/STRMK mutants including Orai1 G98S [81]. *Stim1* I115F GoF mice treated with CIC-39 restored platelet count and prevented abnormal bleeding, offering a potential treatment for thrombocytopenia in the TAM patients [82].

## Concluding remarks

STIM and Orai1 proteins are closely linked to a variety of human diseases affecting multiple organ systems. While some conditions, such as severe combined immunodeficiency (SCID), TAM and ectodermal dysplasia, arise from LoF or GoF mutations, others, including acute pancreatitis and allergic asthma, are characterised by excessive Ca^2+^ influx through otherwise unmutated channels. In many of these diseases, the pathology is not driven by a single homogeneous cell type; rather, multiple cell types, particularly immune cells, play significant roles.

In this context, the widespread distribution of CRAC channels, initially seen as a potential source of severe off-target effects for CRAC channel-focused therapies, could offer advantages. Given that CRAC channel-driven cross-talk between various cell types accelerates disease progression, evident in the positive feedback loops between airway epithelia, diverse lung immune cells and smooth muscle, partial inhibition of CRAC channels across all cell types may exert a supra-linear suppressive effect. This concept has already been demonstrated *in vitro* [83]. By partially inhibiting CRAC channels, the risk of off-target effects is reduced, as is the likelihood of globally impairing CRAC channel activity.

**Perspectives**

- Store-operated CRAC channels serve as a key pathway for Ca²⁺ entry in most cell types and have been implicated in a growing number of human diseases. Understanding how CRAC channels contribute to organ dysfunction across different physiological systems could unveil commonalities across various pathological conditions.
- A recurring theme emerging from studies of seemingly unrelated disorders is that CRAC channel activity is altered in multiple cell types within an organ, with tissue-resident immune cells playing a particularly prominent role.
- As a result, targeting CRAC channels represents an appealing therapeutic approach, as it could simultaneously affect various cell types, potentially mitigating the severity of the disease.

## Open Access

This article has been published open access under our Subscribe to Open programme, made possible through the support of our subscribing institutions, learn more here: https://portlandpress.com/pages/open_access_options_and_prices#conditional

## Abbreviations

CAD: channel-activating domain
CRAC: Ca^2+^ release-activated Ca^2+^
ER: endoplasmic reticulum
GoF: gain-of-function
ILC2: innate lymphoid type 2
LoF: loss-of-function
MCU: mitochondrial Ca²+ uniporter
NFAT: nuclear factor of activated T cell
PACs: pancreatic acinar cells
PARs: protease-activated receptors
SARAF: store-operated calcium entry-associated regulatory factor
SCID: severe combined immunodeficiency
SERCA: Sarco-endoplasmic reticulum Ca^2+^ ATPase
SOAR: STIM Orai-activating region
SR: sarcoplasmic reticulum
STIM: Stromal Interaction Molecule
STIM: Stromal Interaction Molecule
STRMK: Stormorken syndrome
TAM: tubular aggregate myopathy
TM: transmembrane
